# Analytic Model for Feature Maps in the Primary Visual Cortex

**DOI:** 10.3389/fncom.2022.659316

**Published:** 2022-02-04

**Authors:** Xiaochen Liu, Peter A. Robinson

**Affiliations:** ^1^School of Physics, The University of Sydney, Sydney, NSW, Australia; ^2^Center for Integrative Brain Function, The University of Sydney, Sydney, NSW, Australia

**Keywords:** orientation selectivity, ocular dominance, receptive field (RF), primary visual cortex (V1), cortical maps

## Abstract

A compact analytic model is proposed to describe the combined orientation preference (OP) and ocular dominance (OD) features of simple cells and their mutual constraints on the spatial layout of the combined OP-OD map in the primary visual cortex (V1). This model consists of three parts: (i) an anisotropic Laplacian (AL) operator that represents the local neural sensitivity to the orientation of visual inputs; and (ii) obtain a receptive field (RF) operator that models the anisotropic spatial projection from nearby neurons to a given V1 cell over scales of a few tenths of a millimeter and combines with the AL operator to give an overall OP operator; and (iii) a map that describes how the parameters of these operators vary approximately periodically across V1. The parameters of the proposed model maximize the neural response at a given OP with an OP tuning curve fitted to experimental results. It is found that the anisotropy of the AL operator does not significantly affect OP selectivity, which is dominated by the RF anisotropy, consistent with Hubel and Wiesel's original conclusions that orientation tuning width of V1 simple cell is inversely related to the elongation of its RF. A simplified and idealized OP-OD map is then constructed to describe the approximately periodic local OP-OD structure of V1 in a compact form. It is shown explicitly that the OP map can be approximated by retaining its dominant spatial Fourier coefficients, which are shown to suffice to reconstruct its basic spatial structure. Moreover, this representation is a suitable form to analyze observed OP maps compactly and to be used in neural field theory (NFT) for analyzing activity modulated by the OP-OD structure of V1. Application to independently simulated V1 OP structure shows that observed irregularities in the map correspond to a spread of dominant coefficients in a circle in Fourier space. In addition, there is a strong bias toward two perpendicular directions when only a small patch of local map is included. The bias is decreased as the amount of V1 included in the Fourier transform is increased.

## 1. Introduction

The aim of this study is to: (i) build a simple and idealized OP-OD map representation of V1 which is based on the local feature detection in OP and OD, and the modeling of the neural interaction between nearby hypercolumns; and (ii) obtain a suitable Fourier domain representation of the local area of OP-OD map with the range of a few hypercolumns, in order to place it in the form required to link it to the neural field theory (NFT) of neural activities and connections in approximately periodic structures such as primary visual cortex (V1).

V1 is the first cortical area that processes visual inputs from the lateral geniculate nucleus (LGN) of the thalamus before projecting output signals to higher visual areas (Hubel and Wiesel, 1961, 1962a, 1972; Garey and Powell, 1967; Hendrickson et al., 1978; Miikkulainen et al., 2005). The feedforward visual pathway from the eyes to V1 involves two main processing steps: (i) light levels at a given spatial location are detected and converted into neural signals by the retina ganglion cells; and (ii) the neural signals are transmitted to V1 through the lateral geniculate nuclei (LGN) of the thalamus (Schiller and Tehovnik, 2015). LGN neurons have approximately circular receptive fields with either a central ON region (activity enhanced by light incident there) surrounded by an OFF annulus (activity enhanced by darkness there), or vice versa (Hubel and Wiesel, 1961; De Angelis et al., 1995). In addition, recent studies (Suematsu et al., 2012, 2013) have found that most LGN neurons have elongated receptive fields, which provide considerable orientation bias. The current study focuses on circular LGN receptive fields.

Similarly to other parts of the cortex, V1 can be approximated as a two-dimensional sheet when studying the spatial structure of various feature maps (Tovée, 1996). V1 neurons, which respond to same eye preference or orientation preference, are arranged in columns perpendicular to the cortical surface. Columns do not have sharp boundaries; rather, feature preferences gradually vary across the surface of V1. These maps are overlaid such that a given neuron responds to several features (Hubel and Wiesel, 1962b, 1968, 1974a; Miikkulainen et al., 2005).

**Figure 1:**
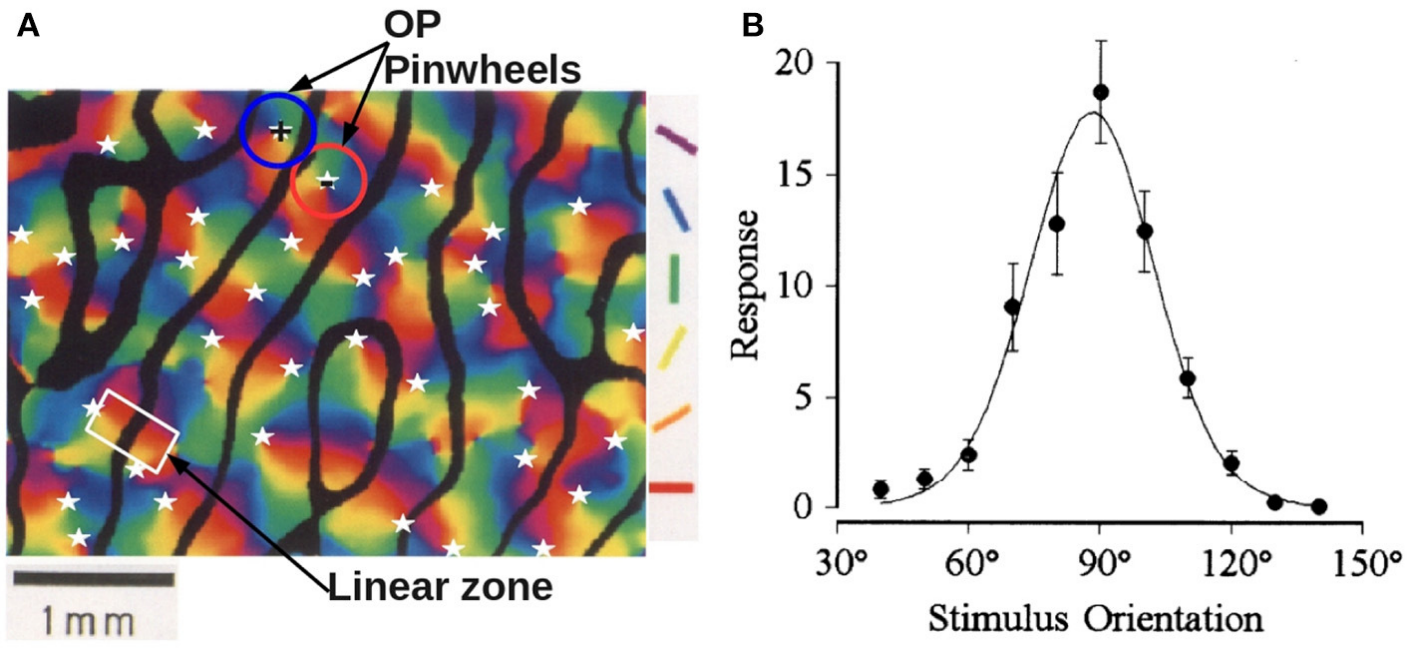
Experimental OP-OD properties. **(A)** Combined OP-OD map of macaque monkey, adapted from Blasdel (1992). The borders of OD stripes are shown in solid black, and singularities (pinwheel centers) are labeled by white stars. Oriented color bars indicate different OPs. The blue and red circles outline examples of positive and negative OP pinwheels, and the white rectangle outlines a linear zone. **(B)** Experimental orientation tuning curve, adapted from Swindale (1998). The preferred orientation angle is around 90°. The dots are the data points, and the solid curve is the fitted tuning curve using a von Mises function.

Two prominent feature preferences of V1 cells are their layout in a combined the OP-OD map, as seen in Figure 1A, which shows an example from experiment (Blasdel, 1992). Hubel and Wiesel (1968) found that neurons that respond preferentially to stimuli from one eye or the other are arranged in alternating bands across layer 4C of V1 in macaque monkeys, and these bands are termed left and right OD stripes. The average OD stripe width in mammals ranges from ~ 0.5 − 1 mm (LeVay et al., 1985; Horton and Adams, 2005; Adams et al., 2007). An OP column, sometimes called an iso-orientation slab, comprises neurons that respond to similar edge orientation in a visual field. Each OP column not only spans several cortical layers vertically, but also extends 25 − 50 μm laterally in monkey. Moreover, OP normally varies continuously as a function of the cortical position, covering the complete range 0° to 180° of edge orientations (Hubel and Wiesel, 1974a, 1977; Obermayer and Blasdel, 1993). Optical imaging reveals that OP columns are quasiperiodic, and are arranged as pinwheels, within which each of the OPs varies azimuthally around a center called a singularity (Bonhoeffer and Grinvald, 1991, 1993; Blasdel, 1992; Swindale, 1996). Furthermore, the OP in each pinwheel increases either clockwise (negative pinwheel) or counterclockwise (positive pinwheel) and most neighboring pinwheels have opposite signs (Götz, 1987, 1988). Examples of positive and negative OP pinwheels are outlined in Figure 1A. The superimposed OD and OP maps have specific relationships, including that: (i) most pinwheels are centered near the middle of OD stripes; (ii) linear zones, which are formed by near-parallel OP columns, usually connect two singularities and cross the border of OD stripes at right angles (Bartfeld and Grinvald, 1992), as highlighted in the white rectangle in Figure 1A. Additionally, various studies (Mitchison, 1991, 1995; Koulakov and Chklovskii, 2001; Chklovskii and Koulakov, 2004) have argued that the appearance of the OP-OD map reflects wiring optimization of local neuron connectivity, in which the distance between neurons with similar feature preference is kept as small as possible.

According to the quasiperiodicity of the feature preference of V1, previous studies (Bressloff and Cowan, 2002; Veltz et al., 2015) suggested that the functional maps of V1 can be approximated by a spatially periodic network of fundamental domains, each of which is called a hypercolumn. Each hypercolumn represents a small piece of V1, which consists of left and right OD stripes with a pair of positive and negative pinwheels in each, so as to ensure the complete coverage of the OP and OD selectivity.

Orientation selectivity plays a primary role in early-stage visual processing. One way to characterize the preferred orientations of a single neuron is to measure the tuning curve from its neuronal response to visual stimuli with various orientations. Figure 1B shows an experimental orientation tuning curve obtained from single unit responses in area 17 of adult cat, with optimal orientation angle of 90° (Swindale, 1998). A typical full width at half maximum (FWHM) of such a curve is ~ 35°.

**Figure 2:**
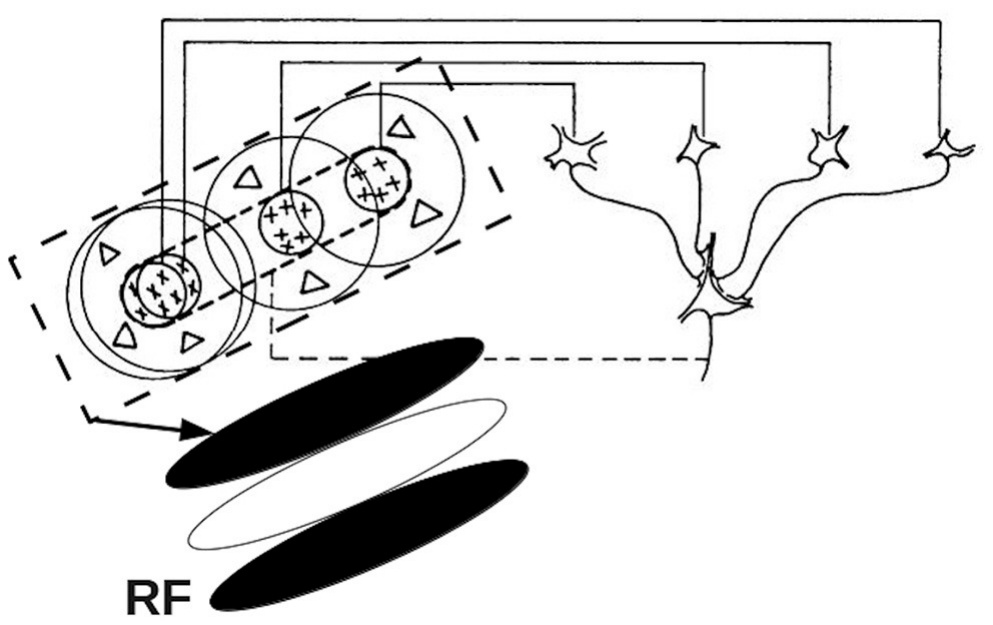
Schematic of the elongated RF of a V1 simple cell, showing the convergence of several LGN RFs into the V1 RF. The four cells in the right half of the figure represents LGN cells with circular ON center, OFF surround RFs. The outputs of these LGN cells project to form the elongated V1 RF shown at the bottom left. Adapted from Hubel and Wiesel (1962a).

The mapping of inputs from the retina to V1 is organized in a retinotopic manner. Visual information in nearby regions within the visual field is projected to neighboring ganglion cells in the retina. This spatial arrangement is maintained through the LGN to V1, where the visual signals are further processed by neighboring V1 neurons. The subregion in the visual field, within which certain features of the visual object tend to evoke or suppress neural firing of a given V1 neuron, is termed the classical receptive field (RF) of that neuron (Hubel and Wiesel, 1962a; Tootell et al., 1982; Skottun et al., 1991; Smith et al., 2001; Schiller and Tehovnik, 2015). The area around the classical RF is referred to as the non-classical or extra-classical RF, in which a stimulus can modulate the responses evoked by stimuli in the classical RF (Allman et al., 1985; Rao and Ballard, 1999; Henry et al., 2013). This paper focuses on the classical RF of V1 simple cells, which respond best to oriented bars. The spatial arrangement of a V1 simple cell RF has separate ON and OFF subareas, which are elongated in a specific orientation, and these subareas relate to the ON and OFF regions of LGN RFs that project to the V1 RF of a given cell. The neuron will be excited when light illuminates the ON subarea, and be depressed when light exposed to the OFF subarea (Hubel and Wiesel, 1968; Mechler and Ringach, 2002). It was first proposed by Hubel and Wiesel (1962a) that the RF of a V1 simple cell can be predicted by a feed-forward model. Specifically, they suggested that RF of a V1 simple cell is formed by combining the circular RFs of several LGN cells to produce an elongated RF with central ON region flanked by OFF regions. Figure 2 shows a schematic of this feed-forward model, which produces a RF with a three-lobed pattern, as shown in the bottom left corner. In addition, several studies have suggested that the lateral intracortical excitatory and inhibitory connectivities from surrounding neurons also play important roles in a cell's orientation tuning and RF formation (Gardner et al., 1999; Ferster and Miller, 2000; Mariño et al., 2005; Finn et al., 2007; Moore IV and Freeman, 2012). Moreover, some studies have discussed the relationship between the size of the RF and the width of the orientation tuning curve (Hubel and Wiesel, 1962a; Lampl et al., 2001). These authors predicted that the width of orientation tuning curve should be inversely associated with the elongation of the RF.

Numerous experiments have used optical imaging or functional magnetic resonance imaging (fMRI) to reveal the spatial structure of the OP-OD map in mammals including humans (Bonhoeffer and Grinvald, 1991, 1993; Bartfeld and Grinvald, 1992; Blasdel, 1992; Obermayer and Blasdel, 1993; Bosking et al., 1997; Yacoub et al., 2008). Additionally, a number of models have been proposed for constructing the OP-OD maps and simulated numerically (Obermayer et al., 1992b; Swindale, 1992; Erwin et al., 1995; Miikkulainen et al., 2005; Bednar, 2009; Barbieri et al., 2012; Stevens et al., 2013). The resulting OP-OD maps obtained from experiments or simulation are only semiregular, as illustrated in Figure 1A. Hence, it usually requires many data points to describe the structure of such maps, which impedes understanding and requires extensive computation to integrate OP-OD maps into models of neural activity in the approximately periodically structured V1. Such models would benefit from a compact approximate analytic representation of the OP-OD map; e.g., to incorporate its structure into existing spatiotemporal correlation analyses of gamma-band oscillations of neural activity using neural field theory (NFT), or to understand propagation via patchy neural connections in V1 (Robinson, 2005, 2006, 2007; Liu et al., 2020).

NFT averages over the properties of many neurons to model brain structure and activity at scales from ~0.1 mm to the whole brain, where it has had many successful comparisons with experiment (Deco et al., 2008). It is thus well placed to analyze activity in V1, including in the wider context of brain activity as a whole (Robinson, 2006), without having to model every neuron individually, which is impractical. However, to do this, it is necessary to obtain a compact Fourier representation of the approximately periodic mm-scale variations of feature sensitivity and to ensure that these are mutually consistent (Robinson, 2006). This formulation then enables the evolution of the corresponding properties of spatially modulated activity evoked by various stimuli to be tracked.

The above issues motivate us to derive a compact analytical representation of the OP-OD map for use in linking the microscale neural activities to the brain dynamics in a larger scale of a few tenths of a millimeter using NFT, and for analysis of properties of OP-OD maps obtained from *in-vivo* experiments or computer simulations. In this study we start with an idealized map that is periodic and regularized. The idealization is suitable in a short scale of a few millimeters, where OD stripes can be approximated as straight and parallel; at longer scales, these stripes deviate in direction over a characteristic correlation length beyond which they tend toward isotropy on average (LeVay et al., 1985; Adams et al., 2007).

To achieve the above aim, we first note that it has long been suggested that oriented visual features such as edges can be detected by the Laplacian operator (Ratliff, 1965; Marr and Hildreth, 1980; Marr and Ullman, 1981; Marr, 1982; Young, 1987). Hence, we approximate the local sensitivity of V1 neuron to the orientation of stimuli by such an operator, which we allow to be anisotropic. This operator incorporates the details of LGN receptive fields, as projected to the cortex, and any anisotropic response at V1, as implemented through local excitatory and inhibitory wiring between neurons. Other edge detectors have been proposed to exist in V1, and a wider range have been suggested in the field of machine vision. These include: (i) the Canny operator, which tracks the maximum gradient points of the Gaussian-smoothed image through a non-maximal suppression process (Canny, 1986); (ii) the Gabor filter that extracts the image features with specific direction and spatial frequencies using a Gaussian modulated sinusoid (Mehrotra et al., 1992); and (iii) difference of Gaussian (DoG) filter, which consists of two Gaussians of opposite sign and different standard deviations (Young, 1987). Some of these filters are suitable for modeling the receptive fields of retinal ganglion cells (e.g., DoG filter) and V1 simple cells (e.g., Gabor filter) (Ringach, 2002; Lindeberg, 2013). It is worth stressing that we are not aiming to incorporate the latest and most sophisticated edge detectors from computer vision; rather, we want a simple operator that can plausibly be implemented in neural tissue. The Laplacian is a suitable such operator, but use of more complex operators would not affect the mutual constraints between OD and OP maps. Secondly, we introduce an RF operator to approximate the anisotropy of the visual region that projects activity to a given V1 simple cell. The combined Laplacian and RF operators yield an overall OP operator at each point, whose parameters can be fitted to yield observed OP tuning widths. Thirdly, we allow the properties of the OP operator to vary across V1 approximately periodically, as described by dominant Fourier coefficients.

The paper is structured as follows: In Section 2, we approximate the hypercolumn with its structure being compatible with the general features of OP-OD map. Meanwhile, it also is compatible with NFT; We describe the local sensitivity to stimulus orientation by applying an anisotropic Laplacian operator to the stimulus. A RF operator is then introduced to project activity from neighboring neurons to a given point. Then, in Section 3, the resulting combined OP operator maximizes the response of V1 neurons to their preferred stimulus orientation and its parameters are adjusted to match experimental tuning curves; Moreover, we Fourier decompose the OP and OD variations on the period of an idealized hypercolumn and investigate the properties of the resulting Fourier coefficients. The results are then applied to compactly represent and analyze OP and OD maps generated from widely used simulation models. Finally, the main findings are discussed and summarized in Section 4.

## 2. Materials and Methods

### 2.1. Hypercolumns and OP-OD Maps

In this section, an approximate OP-OD map within a hypercolumn is proposed, which reproduces the main aspects of observed maps. It is Fourier decomposed in later sections to generate a sparse set of Fourier coefficients that can be used in NFT to study activity across many hypercolumns.

#### 2.1.1. Hypercolumn Arrangement

**Figure 3:**
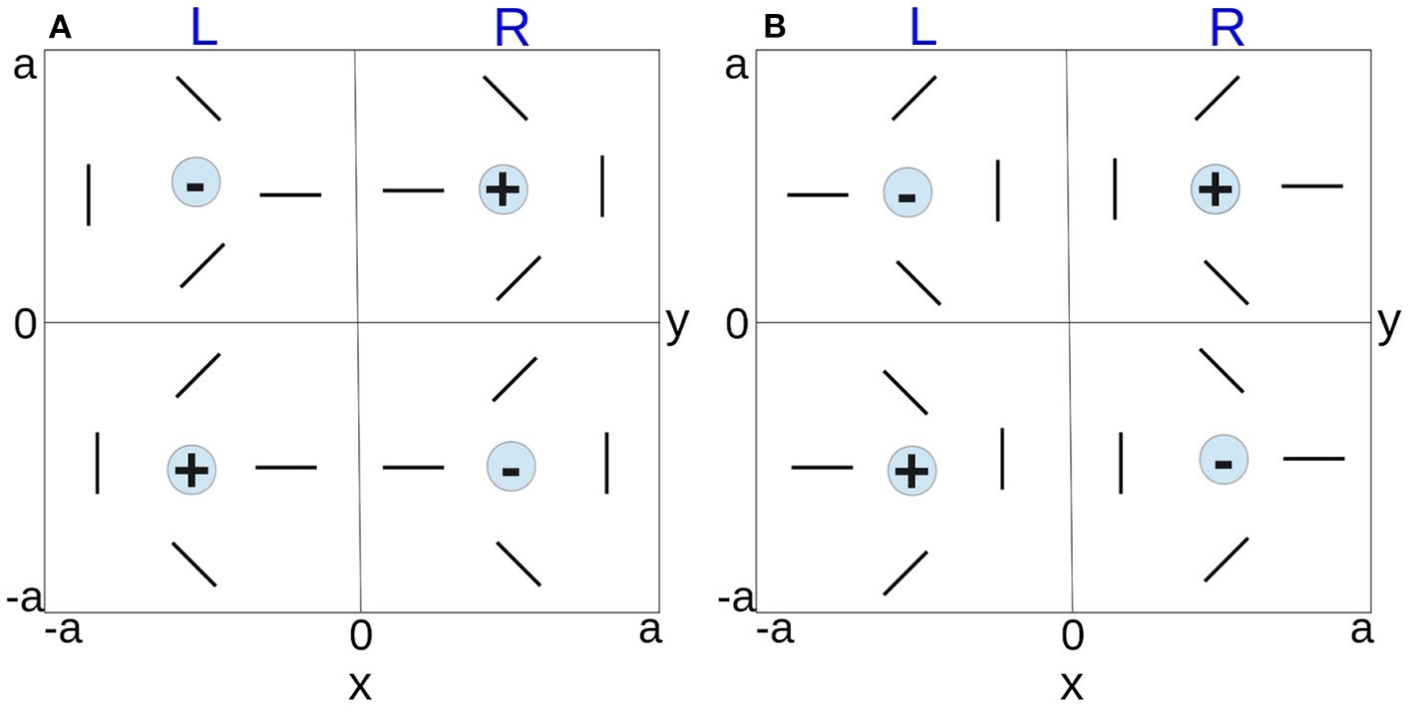
Schematics of possible hypercolumn arrangements. The two vertical bands of each hypercolumn represent the left and right OD stripes. The orientated bars represent the OP within a pinwheel, and the +/− signs indicate the polarity of the pinwheels. **(A)** Pinwheel arrangement I. **(B)** Pinwheel arrangement II.

All feature preferences within a small visual field are mapped to a hypercolumn in V1 (Hubel and Wiesel, 1962b, 1974a; Miikkulainen et al., 2005). Based on the pinwheel model introduced by De Valois and De Valois (1990), we approximate the hypercolumn as a square domain, which consists of left and right OD stripes of equal width; the structure of OD map constrains the variation of OP, such that each OD stripe contains a pair of positive and negative pinwheels for continuous (except at pinwheel centers) and complete feature preference coverage within a hypercolumn. The hypercolumn is consistent with general observations of visual cortical map formation from experimental studies (LeVay et al., 1985; Bonhoeffer and Grinvald, 1991; Bartfeld and Grinvald, 1992; Obermayer et al., 1992a; Obermayer and Blasdel, 1993; Erwin et al., 1995; Müller et al., 2000; Adams et al., 2007), including that: (i) left-eye and right-eye OD stripes are arranged as alternating stripes in V1 of average width ≈1 mm; (ii) OP angles are arranged as pinwheels; (iii) each pinwheel center coincides with the center of its OD band; (iv) OP is continuous at OD boundaries; and (iv) neighboring pinwheels have opposite signs. A + sign denotes a counterclockwise increase of OP around the pinwheel center, whereas a − sign denotes a clockwise increase. According to the rules described above, two distinct arrangements of the hypercolumn are possible, as illustrated in Figure 3, where each hypercolumn is 2*a* wide. Pinwheel arrangement I in Figure 3A is used for further study in later sections, without loss of generality. However, other arrangements produce analogous results due to the fact that the hypercolumn are assumed to be continuous at boundary and periodic across V1, and other hypercolumn arrangements can be obtained by either rotating the pinwheels clockwise/counterclockwise or swapping the left/right column or top/bottom row horizontally or vertically of Figure 3A. Note also that one is free to consider a hypercolumn whose lower left corner is at the center of the current hypercolumn in Figure 3A. Such a hypercolumn has exactly the same structure as in Figure 3B, except for interchange of the left- and right-eye columns.

#### 2.1.2. OD Bands and OP Pinwheel Structure

**Figure 4:**
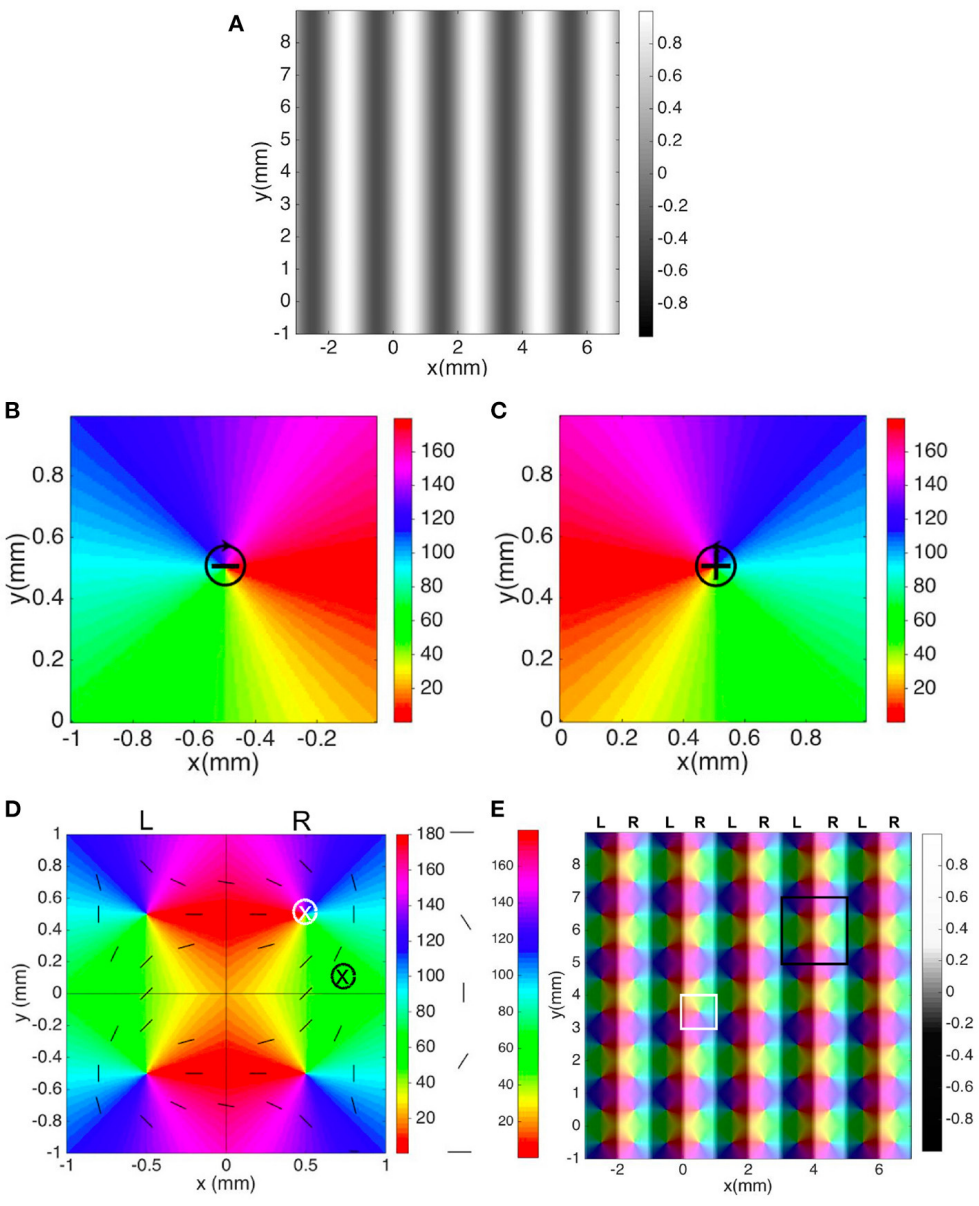
Schematics of visual feature preference maps in V1. **(A)** OD map with left and right OD bands represented by black and white stripes, respectively. The color bar indicates the OD sensitivity, with left-eye preference being negative. **(B)** Negative pinwheel. **(C)** Positive pinwheel. Both pinwheels are 180° periodic and the color bars indicate OP in degrees. **(D)** Hypercolumn. The vertical line divides the hypercolumn into left and right OD bands of equal width, while the horizontal and vertical lines split the hypercolumn into four squares, each containing one OP pinwheel. The white and black crosses mark examples of locations near a pinwheel center and in an iso-orientation domain, and the circle around each cross indicates the characteristic width of the integration region for computing the overall neuron responses in Section 3.2. The short bars highlight the OP at various locations. The 0 and 180° orientations both appear as horizontal bars. The color bar indicates OP in degrees. **(E)** Periodic spatial structure of OP and OD across a small piece of V1 comprising 25 hypercolumns. Black/white stripes indicate left (L) and right (R) OD bands. One pinwheel is outlined in white and one hypercolumn is outlined in black. The left/right color bar indicates OP angle in degrees and OD selectivity.

In our idealized case, we approximate the left and right OD bands as straight parallel stripes. The degree of eye preference is approximated as a sinusoidal function Ω(*x, y*) of cortical position (*x, y*). We define Ω(*x, y*) by subtracting the response to left eye stimuli from the response to the right eye (so positive and negative values indicate R- and L-eye dominance, respectively) and write


(1) $$\begin{array}{l} \Omega \left(x,y\right)=\sin\left(ux+vy\right), \end{array}$$

where


(2) $$\begin{array}{l} u=\frac{\pi }{a}\sin\left(\xi \right),v=\frac{\pi }{a}\cos\left(\xi \right), \end{array}$$

where 2*a* is the period of the OD stripe and ξ is the angle at which the OD stripes measured relative to the *x*-axis, which is set to 90° here (stripes are parallel to the *y* axis). Figure 4A shows the resulting OD map with left/right OD bands represented by black and white stripes, respectively; binocular cells tend to be located near the boundaries between stripes. We note that the exact profile of OD need not be sinusoidal, but this approximation suffices for the present purpose of examining the links between OD and OP maps.

Our model also approximates the OP as a function of cortical location within each hypercolumn. The spatial coordinates of the hypercolumn are set by placing the origin of the coordinates at the center of the hypercolumn, whose boundaries are at *x* = ±*a* and *y* = ±*a*, as shown in Figure 3. The four pinwheels in a single hypercolumn are modeled by first generating the right-top pinwheel, and other pinwheels are produced by mirroring the right-top pinwheel across the *x*-axis, the *y*-axis, and then both. When generating the right-top pinwheel, the *x* and *y* coordinates range from 0 to *a* and the OP angle φ(*x, y*) at each cortical position (*x, y*) is approximated by the inverse tangent function defined as


(3) $$\begin{array}{l} \varphi \left(x,y\right)=\frac{1}{2}\{\begin{array}{ll} \arctan\left(\frac{y-y_{0}}{x-x_{0}}\right), & x>x_{0},y>y_{0}\\ \frac{\pi }{2}, & x=x_{0},y>y_{0}\\ \arctan\left(\frac{y-y_{0}}{x-x_{0}}\right)+\pi , & x<x_{0},y>y_{0}\\ \frac{3\pi }{2}, & x=x_{0},y<y_{0}\\ \arctan\left(\frac{y-y_{0}}{x-x_{0}}\right)+2\pi , & x>x_{0},y<y_{0} \end{array} \end{array}$$

where (*x*_0_, *y*_0_) = (*a*/2, *a*/2) is the center of the right-top pinwheel. The 1/2 coefficient in front of the inverse tangent functions is to make the range of φ(*x, y*) to be 0° to 180°.

The negative and positive pinwheels on the top half of hypercolumn are illustrated in Figures 4B,C. Figure 4D shows a hypercolumn containing four pinwheels. As mentioned previously, the OP and OD features are approximated as continuous and periodic for the moment, so V1 can be approximated as lattice of hypercolumns. Thus, we can construct an array of our approximated hypercolumns to represent a piece of V1, which is shown in Figure 4E. In such an array, the OP structure resembles maps reconstructed from *in-vivo* experiments (e.g., Figure 1A), although the OD stripes are approximated as straight here (Bonhoeffer and Grinvald, 1991, 1993; Blasdel, 1992; Obermayer and Blasdel, 1993).

### 2.2. OP Operator

In this section, we derive analytic representations for the OP of V1 neurons. Firstly, we adopt an anisotropic Laplacian operator to describe the local response of the system to an edge, which can include both the near-isotropic response of the LGN plus any anisotropy introduced by local wiring in V1. We also introduce the RF operator that describes the projection of activity from nearby neurons in an anistropic surrounding region. The combination of these two operators gives an overall OP operator, whose parameters we fit to match experimental tuning curves. Again, we stress that we do not adopt the latest edge detection operator from machine vision; rather, we seek a simple operator that can detect the edges while also be plausibly instantiated by the neural wiring of simple cells. In any event, the precise form operator chosen does not affect the relationship between OP and OD maps, so other variants could be used without affecting our core arguments (but specificity of OP would be affected, in general).

#### 2.2.1. Anisotropic Laplacian (AL) Operator

**Figure 5:**
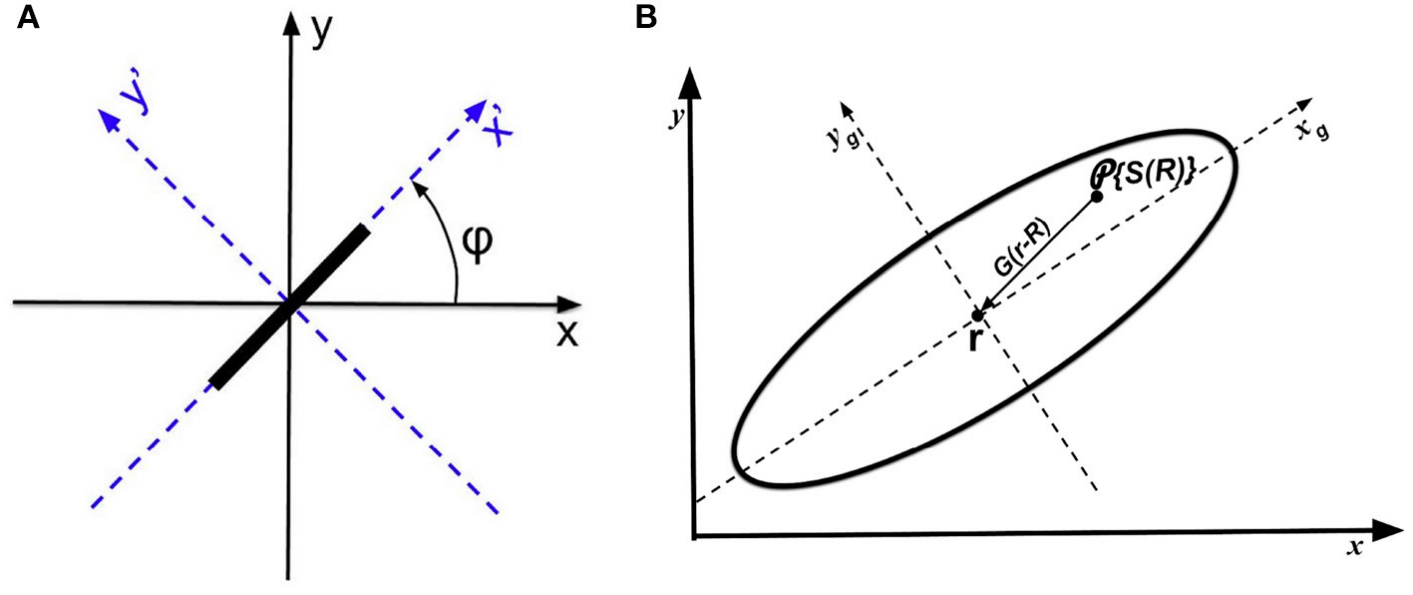
Schematics of coordinates and operators. **(A)** Coordinates used to analyze the anisotropic Laplacian operator. Original axes *x* and *y* are shown in solid, while the rotated axes *x*′ and *y*′ are dashed. The input stimulus is a bar oriented at angle φ. **(B)** Operators leading to the OP response at cells at **r** on the cortex. An oriented bar is mapped to locations **R** in the receptive field, which projects to **r** via the anisotropic weight function *G*(**r** − **R**) indicated by the solid elliptic contour. The local anisotropic Laplacian operator P then acts at **r**. The arrow shows how the neural response are project to measurement point **r** via the weight function. The *x*_*g*_ and *y*_*g*_ are the major and minor axis of the weight function, respectively.

In computer vision, edges with different orientation can be detected by linearly combining the second-order partial derivatives of the input (Marr and Hildreth, 1980; Marr, 1982; Torre and Poggio, 1986). Similarly, we can model the local sensitivity of V1 simple cells to stimulus orientation, by calculating the weighted sum of the second-order partial derivatives of activity projected from the oriented bar stimuli, using rotated axes for simplicity. Figure 5A shows an original *x* − *y* coordinate system, and *x*′ − *y*′ axes obtained by rotating the original axes by an OP angle φ that is the angle of an oriented bar.

A short bar in an image gives rise to localized, anisotropic intensity changes (Torre and Poggio, 1986). In the rotated coordinates, this 2-dimensional intensity change can be detected by the weighted second order partial derivatives in the *x*′ and *y*′ directions. Hence, we define an anisotropic Laplacian operator P as a weighted linear combination of the second order partial derivatives:


(4) $$\begin{array}{l} P=a^{2}\frac{\partial^{2}}{\partial {x{}^{\prime }}^{2}}+b^{2}\frac{\partial^{2}}{\partial {y{}^{\prime }}^{2}}, \end{array}$$

where the rotated coordinates satisfy


(5) $$\begin{array}{l} x^{\prime }=x\cos\varphi +y\sin\varphi , \end{array}$$

(6) $$\begin{array}{l} y^{\prime }=-x\sin\varphi +y\cos\varphi , \end{array}$$

where the OP φ ranges from 0 to π, and *a*^2^ and *b*^2^ are constants.

Taking the Fourier transform of both sides of Equation (4) yields


(7) $$\begin{array}{l} P\left(\text{k}\right)=-a^{2}k_{x^{\prime }}^{2}-b^{2}k_{y^{\prime }}^{2}, \end{array}$$

where


(8) $$\begin{array}{l} k_{x^{\prime }}^{2}={\left(k_{x}\cos\varphi +k_{y}\sin\varphi \right)}^{2}, \end{array}$$

(9) $$\begin{array}{l} k_{y^{\prime }}^{2}={\left(-k_{x}\sin\varphi +k_{y}\cos\varphi \right)}^{2}. \end{array}$$

Substituting Equations (8) and (9) into Equation (7) and then performing an inverse Fourier transform yields


(10) $$\begin{array}{r} P=\left(a^{2}\cos^{2}\varphi +b^{2}\sin^{2}\varphi \right)\frac{\partial^{2}}{\partial x^{2}}+\left(a^{2}-b^{2}\right)\sin\left(2\varphi \right)\frac{\partial^{2}}{\partial x\partial y}\\ +\left(a^{2}\sin^{2}\varphi +b^{2}\cos^{2}\varphi \right)\frac{\partial^{2}}{\partial y^{2}}. \end{array}$$

Since the stimulus *S* is oriented along the *x*′ axis, we would need more weight on ∂^2^/∂*y*′^2^ than on ∂^2^/∂*x*′^2^ to have a maximal response to the desired orientation; this implies that *b*^2^ ≥ *a*^2^.

#### 2.2.2. Receptive Field (RF) Operator

Previous studies (Hubel and Wiesel, 1977; Toth et al., 1997; Troyer et al., 1998; Ferster and Miller, 2000; Schummers et al., 2002; Mariño et al., 2005; Finn et al., 2007) have suggested that the orientation tuning of V1 neuron is altered by the excitatory (inhibitory) inputs from locally connected neighboring neurons (either from the same orientation column or from neighboring columns). Thus, we now introduce an RF operator to model the anisotropic RF that projects to V1 cells. This consists of a weight function *G*(**R** − **r**), which describes the strength of the neural projection from locations **R**, where input stimuli are mapped to V1, to a cell at location **r** whose OP is being approximated. Figure 5B shows a schematic of the RF operator on a piece of cortex, indicating both the location **r** and locations **R** whose activity projects to **r**.

We approximate *G*(**r** − **R**) as an anisotropic Gaussian function whose long axis is oriented at the local OP φ at **R** (Jones and Palmer, 1987). If **R** = (*x*_*R*_, *y*_*R*_) and **r** = (*x, y*), we have


(11) $$\begin{array}{l} G\left(\text{r}-\text{R}\right)=\frac{1}{2\pi \sigma_{x}\sigma_{y}}\exp\left[-\frac{1}{2}\left(\frac{x_{g}^{2}}{\sigma_{x}^{2}}+\frac{y_{g}^{2}}{\sigma_{y}^{2}}\right)\right], \end{array}$$

where


(12) $$\begin{array}{l} x_{g}=\left(x-x_{R}\right)\cos\varphi +\left(y-y_{R}\right)\sin\varphi , \end{array}$$

(13) $$\begin{array}{l} y_{g}=-\left(x-x_{R}\right)\sin\varphi +\left(y-y_{R}\right)\cos\varphi . \end{array}$$

The appropriate width of *G*(**r** − **R**) along the *x*_*g*_ axis is determined by three factors: (i) the approximate RF size near the fovea measured from experiments. Previous studies (Hubel and Wiesel, 1974b; Dow et al., 1981; Keliris et al., 2019) yielded an RF size of ≈0.082° at the eccentricity of 1° in macaque Monkey. We then transform the RF size in visual degree to the corresponding cortical size in mm by adopting the magnification factor from Horton and Hoyt (1991), where the magnification factor *M* (expressed in mm per degree) in monkey is approximated as


(14) $$\begin{array}{l} M=\frac{12}{E+0.75}, \end{array}$$

where *E* is the eccentricity in degrees. The corresponding cortical RF size is then ~0.56 mm; (ii) the width of the weight function should ensure an approximate 30° fall off from the measuring neuron's maximum response when it is activated by its optimal orientation (De Valois et al., 1982; Swindale, 1998; Ringach et al., 2002; Gur et al., 2005; Moore IV and Freeman, 2012); (iii) In local connections, the typical axonal projection range of a V1 neuron lies between 0.18 and 0.3 mm (Lund, 1973; Blasdel et al., 1985; Fitzpatrick et al., 1985; Vanni et al., 2020). In order to satisfy all the three factors mentioned above, we choose σ_*x*_ = 0.18 mm.

#### 2.2.3. Combined OP Operator

Here we combine the anisotropic Laplacian and RF operators from above to obtain an overall OP operator and adjust its parameters to match experimental OP tuning curves.

The input that reaches location **R** on the cortex is approximated by applying the AL operator to the stimulus *S* (i.e., P{*S*(**R**)} in Figure 5B), so that the local orientation sensitivity is picked out, while the weight function *G*(**R** − **r**) determines how much response from locations **R** are projected to **r**. Hence, the response at **r** can be approximated by convolving the weight function with the OP operator on the stimulus at different location **R**. It can be written as


(15) $$\begin{array}{l} I\left(\text{r}\right)=\int G\left(\text{r}-\text{R}\right)P\left\{S\left(\text{R}\right)\right\}\;d\text{R}. \end{array}$$

In the Fourier domain, the convolution theorem yields


(16) $$\begin{array}{l} I\left(\text{k}\right)=G\left(\text{k}\right)P\left(\text{k}\right)S\left(\text{k}\right). \end{array}$$

where the algebraic function P(**k**) is defined in Equation (7). We can thus reverse the order of P(**k**) and *G*(**k**) on the right hand side of Equation (16) and inverse Fourier transform to obtain


(17) $$\begin{array}{l} I\left(\text{r}\right)=\int P\left\{G\left(\text{r}-\text{R}\right)\right\}S\left(\text{R}\right)d\text{R}. \end{array}$$

Hence, the response at **r** becomes the convolution of a new combined OP operator P{*G*(**r** − **R**)} with the stimulus itself; the resulting operator is a Gaussian-derivative operator. This result agrees with previous studies (Movshon et al., 1978; Graham, 1989), which indicated that V1 simple cell can be modeled as a linear filter and its responses are computed as the weighted integral of the Laplacian-transformed stimulus, with the weights given by the RF pattern.

**Figure 6:**
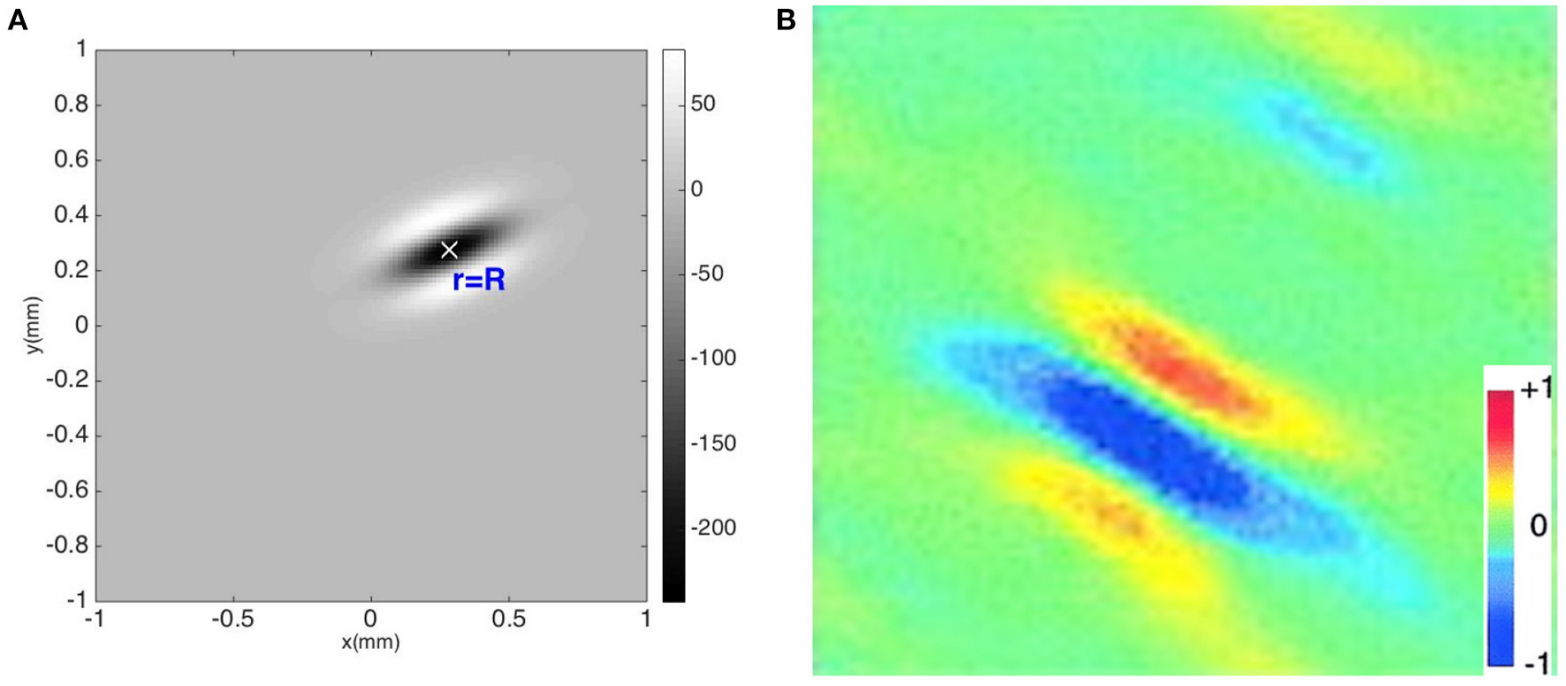
Comparison of theoretical and experimental OP detection. **(A)** OP operator P{*G*(**R** − **r**)} with OP = 22.5°. The color bar indicates the amplitude of the operator. **(B)** RF of macaque monkey V1 simple cell from experiment (Ringach, 2002). The color bar indicates normalized impulse response strength of the neurons.

Figure 6A shows a contour plot of the combined OP operator P{*G*(**r** − **R**)} with a preferred orientation angle of φ = 22.5°. The operator has an elongated three-lobe pattern with its major axis along the direction φ, with an ON center lobe and two OFF side lobes. Figure 6B shows a measured RF of V1 simple cells of macaque monkey (Ringach, 2002), showing that our OP operator closely resembles the experimental one in spatial structure. Some studies have used different OP operators such as Gabor functions, difference-of-Gaussian functions, or Gaussian derivatives to describe the spatial structure of the RF (Young, 1987; Lindeberg, 2013). Second order Gaussian derivatives and difference-of-Gaussian functions are dominated by a central peak flanked by two peaks of opposite sign, and our function shares these features. Gabor functions and functions involving higher Gaussian derivatives, for example, have smaller additional peaks, which are observed in a small fraction of V1 simple cells (De Valois et al., 2000; Ringach, 2002). However, these additional features do not change the OP of the cell and do not affect the results below, which depend on this OP, not the details of how it was detected.

## 3. Results

### 3.1. Angular Selectivity of the OP Operator

The full width at half maximum (FWHM) of the bell-shaped OP angle tuning curve plotted from the neuron response by convolving the RF operator and the stimulus can be used to parameterize the OP selectivity of the OP operator. The parameters are tunable by adjusting the ratio $\sigma_{x}^{2}/\sigma_{y}^{2}$ of the weight function *G*(**r** − **R**) defined in Equation (11), and the ratio *b*^2^/*a*^2^ of the anisotropic Laplacian operator P defined in Equation (10). Hence, we can find the optimal parameter values of the combined OP operator by adjusting its parameters so its tuning curve matches experiment.

**Figure 7:**
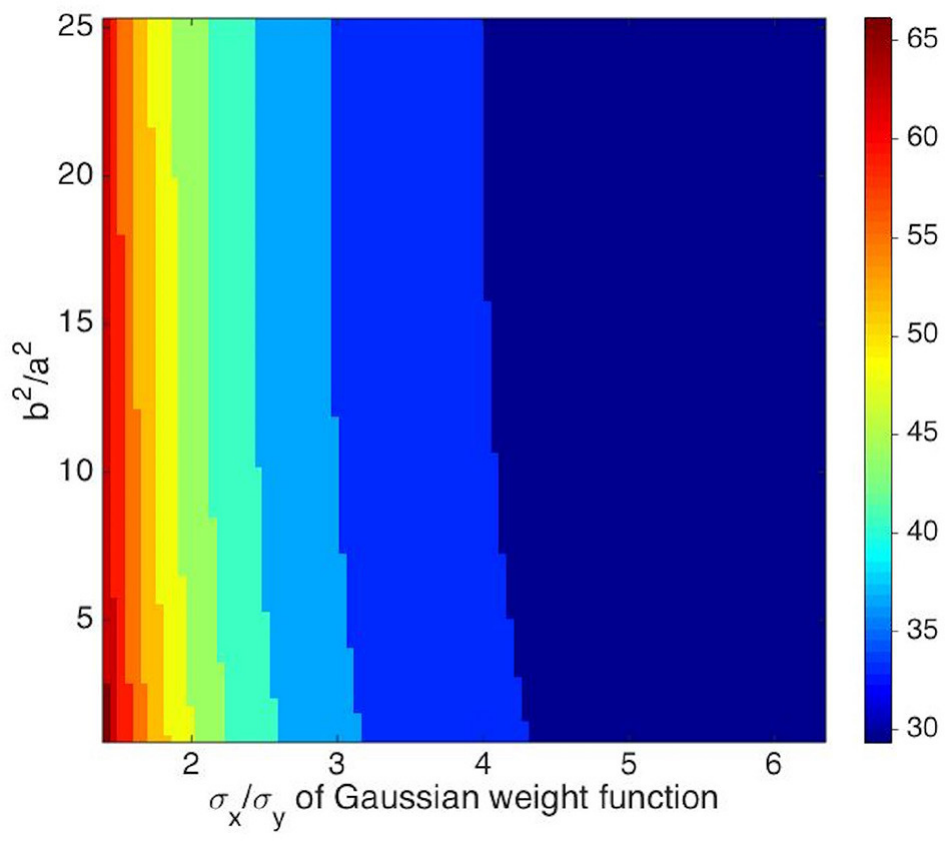
Contour map of the FWHM of the OP tuning curve vs. *b*^2^/*a*^2^, and σ_*x*_/σ_*y*_. The preferred orientation angle at measurement point is 135°. The value of σ_*x*_/σ_*y*_ is given by *x* axis, and the ratio of *b*^2^ and *a*^2^ is given by *y* axis. The color bar represents the FWHM width in degrees.

We first vary the values of *a*^2^ and *b*^2^ while keeping their sum constant by writing


(18) $$\begin{array}{l} a^{2}=\sin^{2}\psi , \end{array}$$

(19) $$\begin{array}{l} b^{2}=\cos^{2}\psi , \end{array}$$

where ψ ranges from 0 to π/4 to ensure *b*^2^ ≥ *a*^2^. We also vary the ratio σ_*x*_/σ_*y*_ from 1.5 to 6.5 to elongate the weight function along the *x*_*g*_ axis defined in Equation (12). Figure 7 shows the resulting contour map of the FWHM vs. *b*^2^/*a*^2^ and σ_*x*_/σ_*y*_. The FWHM varies rapidly with σ_*x*_/σ_*y*_, with sharper tuning as σ_*x*_/σ_*y*_ increases. In contrast, the FWHM only sharpens slightly when *b*^2^/*a*^2^ increases.

**Figure 8:**
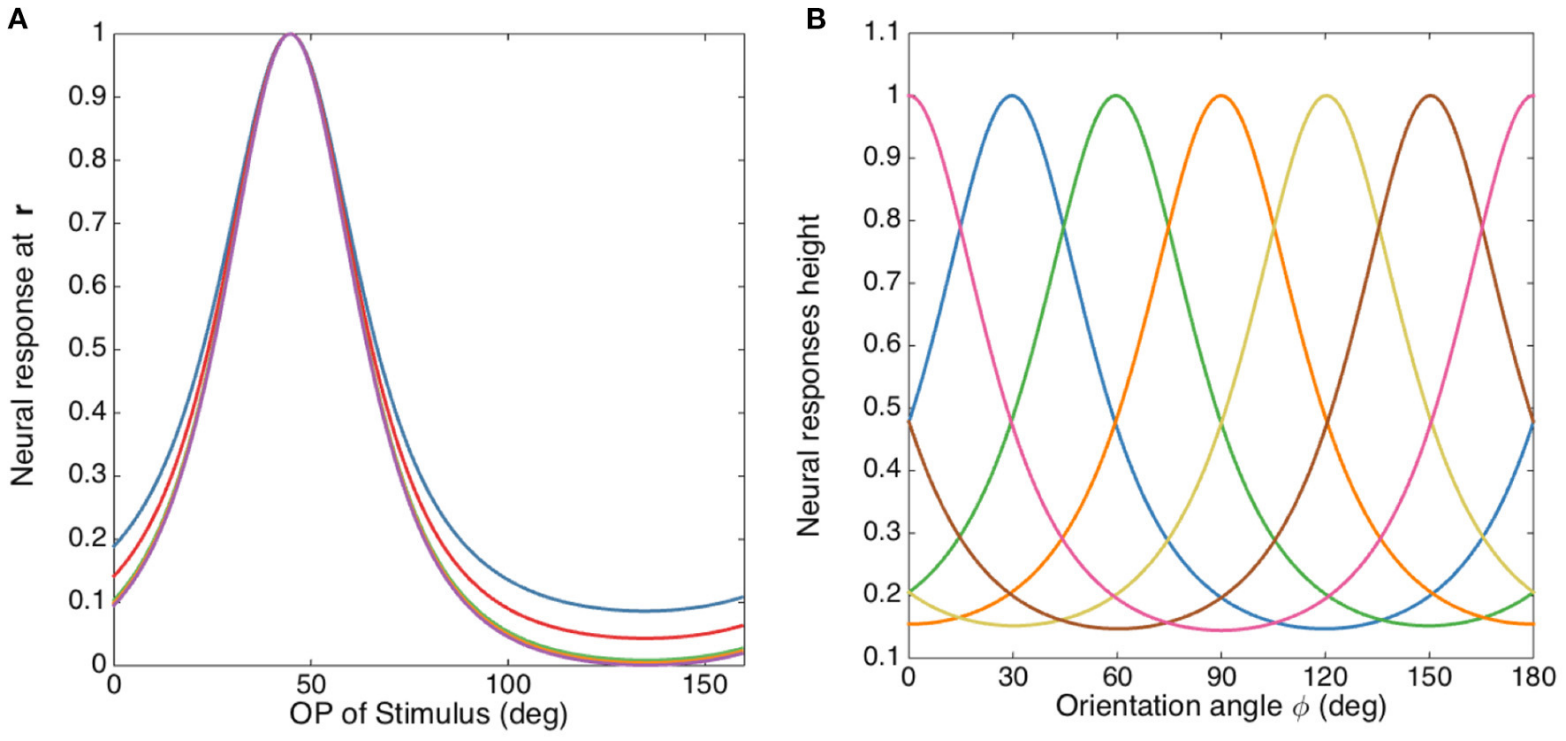
OP tuning curves. **(A)** Normalized tuning curves with *b*^2^/*a*^2^ set to 1 (blue), 2 (red), 10 (green), 20 (yellow), and 100 (purple); and fixing σ_*x*_/σ_*y*_ = 2.5. The preferred orientation angle is set to 45°. **(B)** OP tuning curves with orientation angles 0° (pink), 30° (blue), 60° (green), 90° (orange), 120° (yellow), 150° (brown), and 180° (pink), with σ_*x*_/σ_*y*_ = 2 and *b*^2^/*a*^2^ = 1.

In order to illustrate the insensitivity of the FWHM to *b*/*a* more clearly, Figure 8A shows the normalized tuning curves for fixed σ_*x*_/σ_*y*_ = 2.5, varying *b*^2^/*a*^2^ from 1 to 100. The FWHM decreases by only ~2° when *b*^2^/*a*^2^ changes from 1 to 5, and it does not decrease significantly further for larger *b*^2^/*a*^2^. The reason for this is that the RF operator envelope defined by *G*(**r** − **R**) limits the effective lengths of its three lobes to the Gaussian envelope's characteristic width, so they do not change much when *b*^2^/*a*^2^ increases. This agrees with previous studies, which argued that the OP tuning width of a V1 simple cell varies inversely with the size of its RF (Hubel and Wiesel, 1962a; Lampl et al., 2001). It is also consistent with the Gaussian derivative model proposed by Lindeberg (2013) and Young et al. (2001) for modeling the spatiotemporal RF of V1 cells. Our results also match Hubel and Wiesel's feed-forward model, in which the overall V1 RF results from the net effect of aggregating isotropic LGN RFs via anisotropic connections in V1 (Hubel and Wiesel, 1962a).

The above analysis implies that we can simplify the OP operator by setting *b*^2^ = *a*^2^ because their ratio does not affect the OP tuning width significantly. Then the OP operator P{*G*(**r** − **R**)} becomes the Laplacian of the weight function L{*G*(**r** − **R**)} and the tuning width is controlled by the elongation of the weight function *G*(**r** − **R**).

Previous studies (Pei et al., 1994; Volgushev et al., 2000; Gillespie et al., 2001; Lampl et al., 2001; Goris et al., 2015) suggested that the linear prediction of orientation tuning curves resulting from the spatial structure of receptive field maps (such as the OP operator in the current study) showed a better match with the responses from intracellular membrane potential, rather than the spike activity. The nonlinear effects imposed by the transformation of membrane potential to spikes (i.e., spikes are produced only when potentials is higher than the spike threshold) sharpens the final tuning curve. From the studies mentioned above, the FWHM measured from membrane potentials ranges from 45° for highly selective cells (i.e., with long and narrow RF) to 100° for less selective cells (i.e., with short and wide RF). In this study, we focus on the high selective cells with narrow tuning. Thus, we chose to match our model with the experimental FWHM of 45°, and this corresponds to σ_*x*_/σ_*y*_ ≈ 2. The tuning curves corresponding to this ratio is shown in Figure 8B for various optimal OP.

### 3.2. Tuning Curves vs. Distance From Pinwheel Center

Early experiments showed that neuron populations near pinwheel centers have low mean orientation selectivity (i.e., broad tuning curve), by using optical imaging with voltage sensitive dyes (Blasdel, 1992; Obermayer and Blasdel, 1993), although individual neurons in these regions have good OP selectivity (Bartfeld and Grinvald, 1992; Maldonado et al., 1997; Ohki et al., 2006). We next compute the overall response at a measurement site located at **r**_0_ [i.e., *I*_*OP*_(**r**_0_)] and compare it with experimental results. The overall responses is computed by taking a weighted average of the neural responses around the measurement location by integrating the responses of all the cells with a Gaussian weight function centered at **r**_0_ over the region:


(20) $$\begin{array}{l} I_{OP}\left({\text{r}}_{0}\right)=\int I\left(\text{r}\right)W\left(\text{r}-{\text{r}}_{0}\right)d\text{r}, \end{array}$$

where


(21) $$\begin{array}{l} W\left(\text{r}-{\text{r}}_{0}\right)=\frac{1}{2\pi \sigma_{r}^{2}}\exp\left[-\frac{{\left(\text{r}-{\text{r}}_{0}\right)}^{2}}{2\sigma_{r}^{2}}\right]. \end{array}$$

The width of the weight function is set to 40 μm, to approximate the characteristic width of an OP microcolumn and the experimental range of pinwheel-center effects on OP selectivity (Obermayer and Blasdel, 1993; Maldonado et al., 1997; Ohki et al., 2006; Nauhaus et al., 2008).

**Figure 9:**
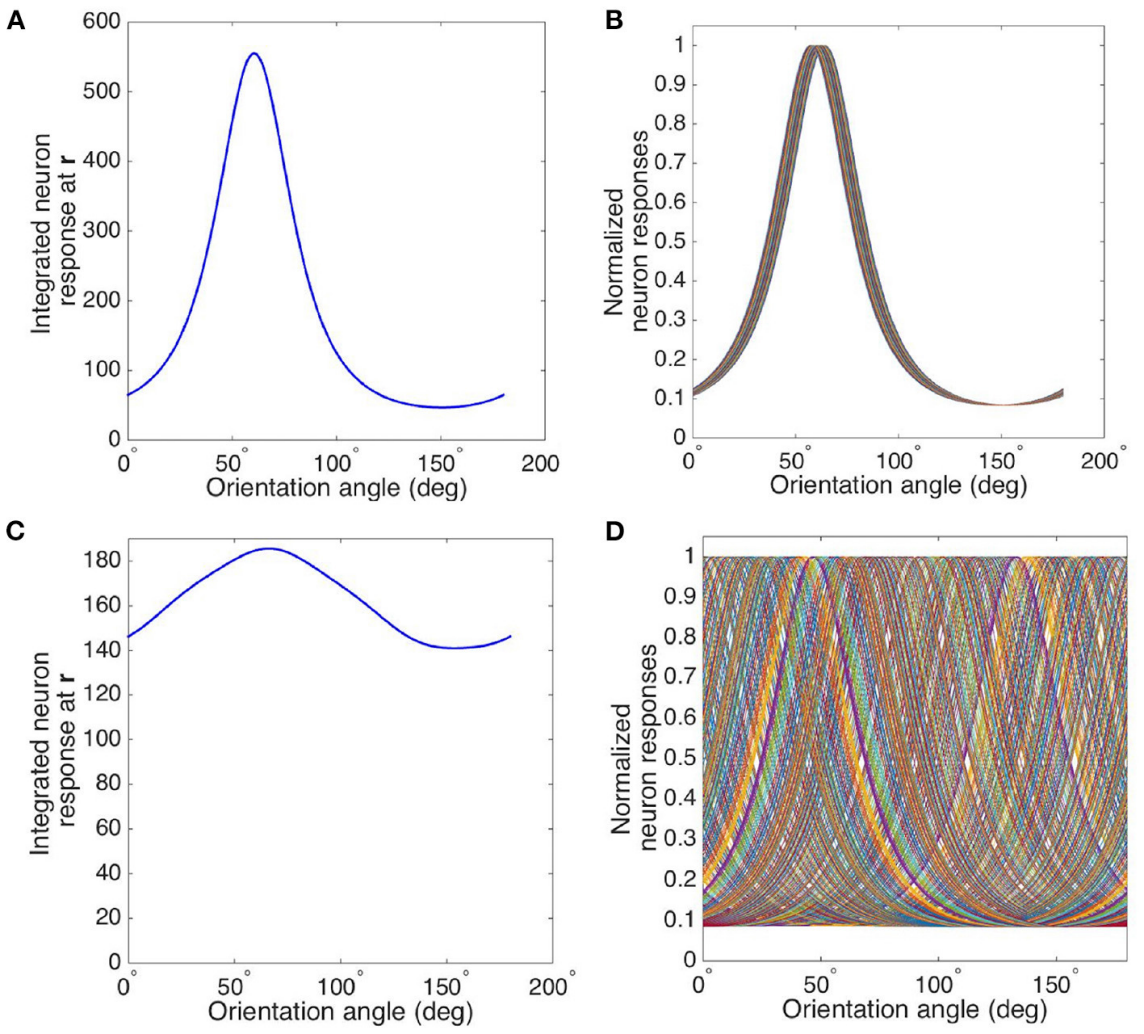
Averaging of OP tuning curves at different distances from a pinwheel center. **(A)** Tuning curve of averaged responses at measurement site located in iso-orientation domain (i.e., marked as black cross in Figure 4D). **(B)** Tuning curves of all the cells surrounding the measurement site within the circular region in iso-orientation domain. **(C)** Tuning curve of averaged responses at measurement site located near pinwheel center (i.e., marked as white cross in Figure 4D). **(D)** Tuning curves of all the cells surrounding the measurement site within the circular region near pinwheel center.

We consider two cases of tuning curves for measurement sites with different locations in the hypercolumn, one near a pinwheel center and another one in an iso-orientation domain. These locations are marked with crosses in Figure 4D, and the circle around each cross indicates the characteristic width of the weight function in Equation (21). Figure 9A shows the resulting tuning curve of the overall responses of a measurement site located in an iso-orientation domain (i.e., the location marked with black cross in Figure 4D) with preferred orientation ≈60°. It is sharply peaked at the preferred angle with FWHM ≈41°. In Figure 9B, we plot the tuning curves for an array of cells that are around the measurement site within the circular region. Since all the cells are located in the iso-orientation domain, they have very similar orientation preferences and tuning curves. The average response of cells near the pinwheel center is plotted in Figure 9C, it is much broader than the tuning curve shown in Figure 9A due to the fact that we average the responses from neurons with a wide range of OPs, as shown in Figure 9D. Our predictions agree with the experimental results (Bartfeld and Grinvald, 1992; Maldonado et al., 1997; Ohki et al., 2006), who found that individual neurons near the pinwheel center are just as orientation selective as the ones in the iso-orientation domain, and the overall broadly tuned response seen by Blasdel and Salama (1986) for example is the averaged response of nearby cells with a wide range of OPs.

**Figure 10:**
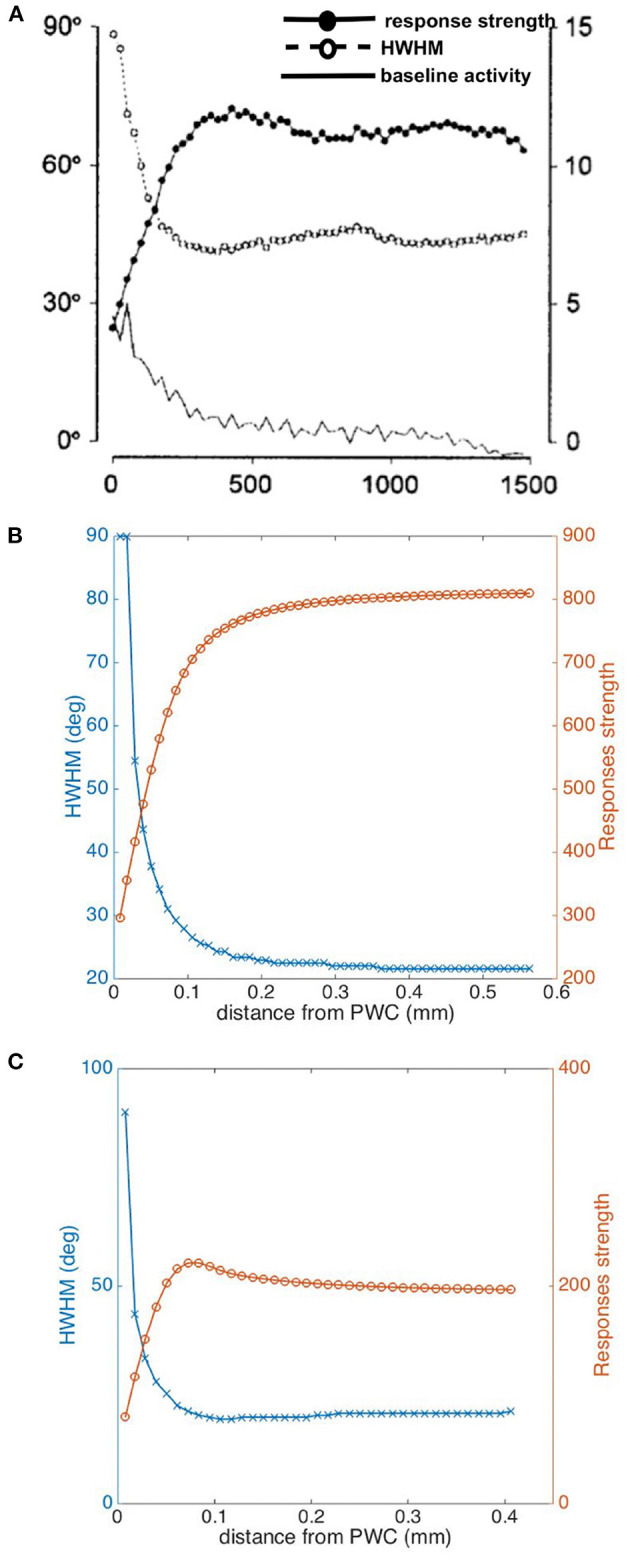
Dependence of tuning properties on distance from a pinwheel center. **(A)** Experimental HWHM and response strength vs. distance in μm from pinwheel center from Swindale et al. (2003), averaged over 13 pinwheels. The filled circles shows the response strength in arbitrary units, while the open circles show the HWHM in degrees and the bottom-most curve shows the baseline activity. **(B)** Predicted HWHM vs. distance (blue) and Responses strength vs. distance (orange) from pinwheel center, by using a Gaussian function as weight function for averaging the responses. **(C)** Predicted HWHM vs. distance (blue) and response strength vs. distance (orange) from pinwheel center, by using a Mexican hat function as weight function for averaging the responses.

We have also investigated how the tuning width and response strength vary with distance from the pinwheel center, and compare them with experiment in Figure 10. We calculate half width at half maximum (HWHM) here, in order to be consistent with the experimental plots. As expected, our predicted HWHM decreases when moving away from the pinwheel center to an iso-orientation domain, while the response strength increases with distance, as seen in Figure 10B. Both results match the experimental findings shown in Figure 10A, except the experimental HWHM in iso-orientation domain is wider than ours; However, our plots do not reproduce the overshoot and dip in the responses strength and HWHM curves, respectively, shown in Figure 10A. In order to achieve a better fit to the experimental data, we thus try the Mexican hat function,


(22) $$\begin{array}{l} W_{mex}\left(\text{r}-{\text{r}}_{0}\right)=\left[1-\frac{{\left(\text{r}-{\text{r}}_{0}\right)}^{2}}{2\sigma_{r}^{2}}\right]\exp\left[-\frac{{\left(\text{r}-{\text{r}}_{0}\right)}^{2}}{2\sigma_{r}^{2}}\right], \end{array}$$

as the weight function to average the responses, and the resulting plots are shown in Figure 10C. Overshoot and dip features are visible in this case, implying a better match. Thus, it is potentially possible to deduce the shape of the weight function from experimental results such as these, but detailed exploration is beyond the scope of the present paper.

### 3.3. Fourier Analysis of the OP-OD Map

We seek a representation of the OP map in the Fourier domain so that we can apply it to compactly represent experimental data and to study spatiotemporal neural activity patterns in periodic V1 structures using NFT, which requires such Fourier coefficients as input. Thus, in this section, we decompose the OP-OD map of the hypercolumn that is defined in Section 2.1 in the Fourier domain, derive the two sets of the Fourier coefficients that represent the spatial frequency components of the OD and OP map structure respectively, and discuss their properties. We also determine the least number of Fourier coefficients we need in NFT analysis while maintaining the essential features of the OP-OD map. This is achieved by reconstructing the OP-OD map with a subset of the coefficients using the inverse Fourier transform.

**Figure 11:**
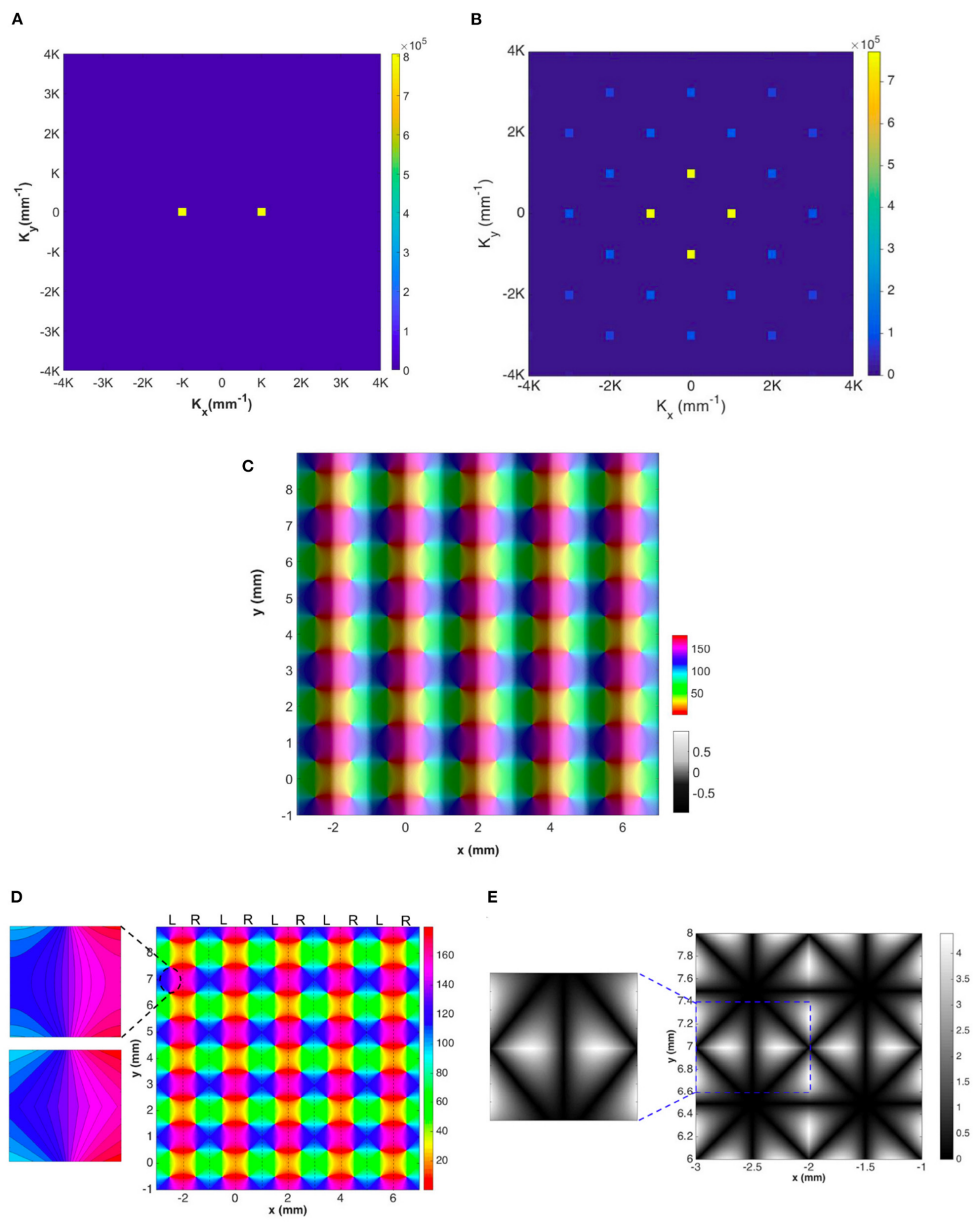
Representations of the OD-constrained OP-OD map for a lattice of 25 hypercolumns. **(A)** Magnitude of Fourier coefficients of the OD map. **(B)** Magnitude of Fourier coefficients of the OP map after applying the operator O. In both **(A,B)**, each square on the figure represents one spatial mode **K**, and the color bar indicates its magnitude. **(C)** Reconstructed OP-OD map using the two sets of dominant Fourier coefficients shown in **(A,B)**. **(D)** Reconstructed OP map with the two squares on the top-left are the zoomed-in patches of the reconstructed (top) and the original (bottom) lattice. These are extracted from the same location that is marked by dashed oval. The color bar indicates the OP in degrees. **(E)** Absolute differences between the original hypercolumn OP in Figure 4E and the reconstructed one. The square on the left shows a zoomed-in patch that is extracted from the location marked by blue dashed-line. The color bar indicates the difference in degrees.

We analyze the OD map shown in Figure 4A by directly performing 2D Fourier transform on the map. The magnitude plot of the resulting Fourier coefficients has 2-fold symmetry and is shown in Figure 11A. As the OD map is modeled by sinusoidal function, it only has two dominant **K** modes, which are located at **k** = (±*K*, 0), where *K* = π/*a* and 2*a* is the period of the OD stripe.

We analyze the OP map in the hypercolumn by first applying a spatial operator O to the map, with O defined as


(23) $$\begin{array}{l} O=\exp\left[i2\varphi \left(x,y\right)\right]. \end{array}$$

This operator preserves the structure and periodicity of the OP-OD map and allows us to avoid the spurious discontinuities between 0° and 180° orientations, which actually correspond to the same stimulus orientation (Swindale et al., 1987). This is important because representation of such discontinuities would require use of high spatial frequencies, and thus many Fourier coefficients. We then perform a 2D Fourier transform on the resulting map, which yields a sparse set of Fourier coefficients.

Figure 11B shows the magnitude of the Fourier coefficients of a lattice of 5 × 5 hypercolumns (i.e., Figure 4E). We note that: (i) the coefficients have 4-fold symmetry, and the four **K** modes with lowest spatial frequency are dominant; and (ii) the lowest **K** modes are located at (±π/*a*, 0) and (0, ±π/*a*), where 2*a* is the width of hypercolumn. The strong bias toward vertical and horizontal directions in the Fourier domain is introduced by the regularized arrangements of the map, such that the OD stripes are straight and parallel to each other and the OP pinwheels are approximated as lying on a square grid. This idealization is suitable for OP-OD maps at short scales of a few millimeters; as larger areas of V1 are included, the direction of OD stripes increasingly varies until overall near-isotropy is attained (LeVay et al., 1985; Niebur and Wörgötter, 1994; Adams et al., 2007). We stress that local isotropy of the map is not possible unless no OP-OD map structure exists to break that symmetry.

One of our main aims in finding these Fourier coefficients of the OP-OD map is to incorporate the OP map structure into the patchy propagator theory introduced to treat periodic V1 structure in previous studies (Robinson, 2006, 2007; Liu et al., 2020). We want to use as few coefficients as possible to simplify computation, while preserving the essential OP-OD structure. In order to test how well a small subset of Fourier coefficients can approximate the OP-OD map, we reconstruct the lattice of hypercolumns from these coefficients and compare it to the original one. To reconstruct the OD map, we perform the inverse Fourier transform including only the two Fourier coefficients with the lowest spatial frequency. Likewise, when reconstructing the OP map, we perform the inverse Fourier transform on the small set of coefficients with lowest spatial frequency. The resulting complex data represents the values that have been transformed from the OP angles after applying the operator O defined in Equation (23). We then transform the complex data back to OP angles via


(24) $$\begin{array}{l} e^{i\varphi \left(x,y\right)}=\cos\left[\varphi \left(x,y\right)\right]+i\sin\left[\varphi \left(x,y\right)\right], \end{array}$$

whence


(25) $$\begin{array}{l} \varphi \left(x,y\right)=\tan^{-1}\left[\frac{\sin\varphi \left(x,y\right)}{\cos\varphi \left(x,y\right)}\right]. \end{array}$$

We find that the **K** modes with lowest spatial frequency (the yellow squares in both Figures 11A,B) suffice to reproduce the main features of the hypercolumn lattice, and the reconstructed map with combined OP and OD is shown in Figure 11C, which is very similar to the original one (i.e., Figure 4E) except that the absence of higher modes leads to differences of up to 4° in OP and some angular contours of the OP map are smoother than in the original map. This detail is shown in the top left frame of Figure 11D, which is to be compared with the frame below it, which is from the same part of the original lattice.

Figure 11E shows the absolute differences between the original OP map in one hypercolumn and the reconstructed one. The square on the left is a zoomed-in patch that is marked by the dashed square, and it is extracted in the same location as we do for the zoomed-in patch in Figure 11D. The largest difference is ≈4.5° near the edges of each pinwheel. Nevertheless, the basic structure and periodicity of the hypercolumn are all preserved in the reconstructed lattice. Thus, we can conclude that the **K** modes with lowest spatial frequency in Figures 11A,B are sufficient for incorporating the OP-OD map structure into NFT computations.

**Figure 12:**
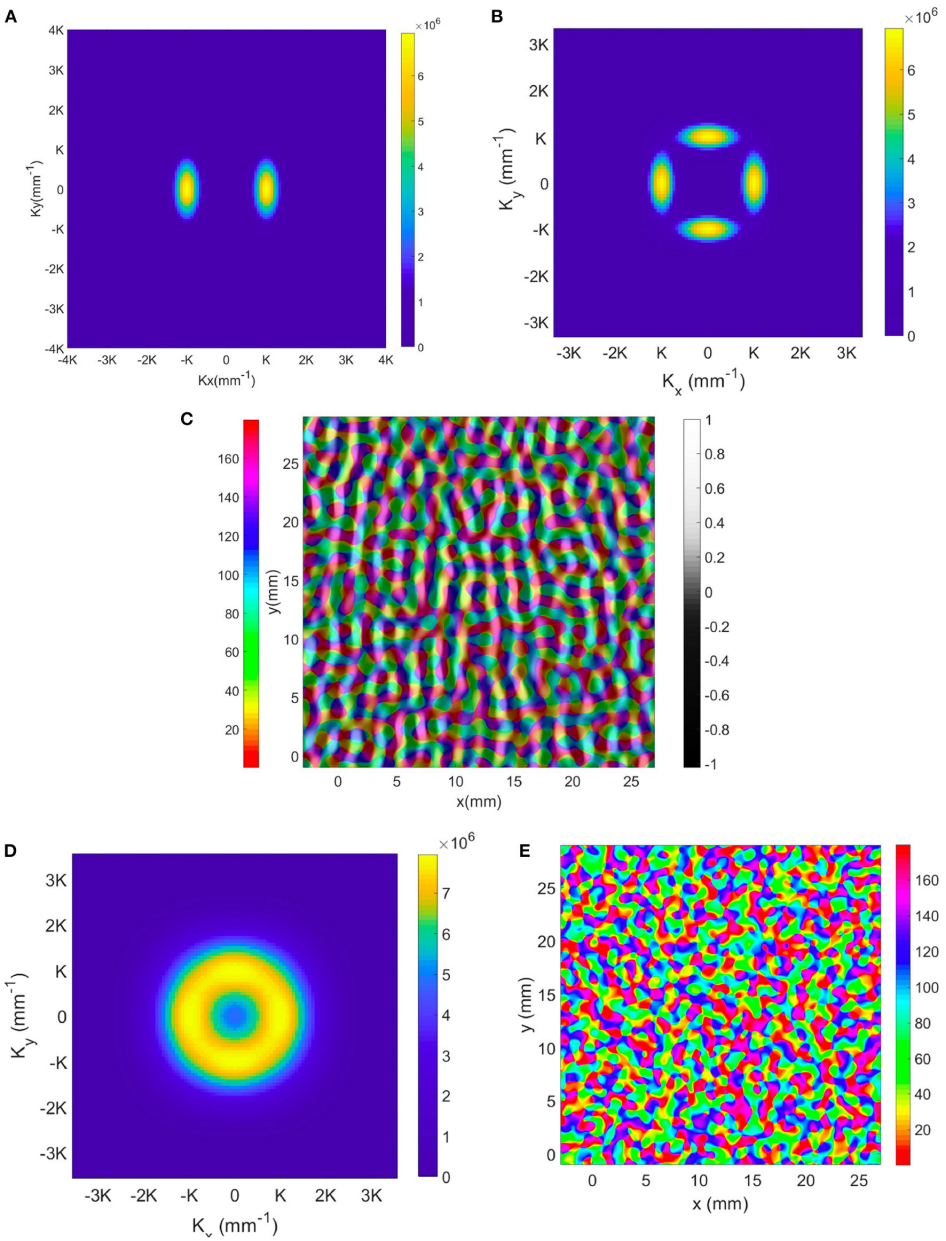
Illustrative OP-OD map with a distribution of **K** in Gaussian envelopes centered on principal modes of the idealized case. **(A)** Magnitude plot of OD **K** modes showing 2-fold symmetry. The color bar indicates the magnitude. **(B)** Magnitude plot of OP **K** modes showing 4-fold symmetry. **(C)** Illustrative OD-constrained OP map using the sets of **K** modes shown in **(A,B)**. The left color bar indicates the OP angle in degrees. **(D)** Magnitude plot of **K** modes with approximate circular symmetry. The color bar indicates the magnitude. **(E)** Reconstructed map using set of **K** modes shown in **(D)**. The color bar indicates the OP angle in degrees.

In V1, the arrangement of hypercolumns is not as regular as our idealization above. To model the development of irregularities in the actual structure, one would simulate cortical plasticity using NFT driven by an ensemble of input stimuli with various features using the present OP operators and OP-OD constraints. As in other branches of physics where local order gives way to long-range disorder, one would expect that randomness in the ensemble would break the local symmetries over some correlation length, giving rise to disorder in the OP-OD map across V1 as a whole. For the moment, though, we can illustrate such situations, and show that the present analysis has sufficient flexibility to describe them, by adding spatial modes around the dominant **K** modes. Our approach for illustrating the effects of disorder in a larger-scale OP map is to add **K** modes around the previous 2 (for OD) and 4 (for OP) lowest spatial frequency modes, with magnitude *M*_*k*_ that is defined by Gaussian envelopes in *K*_*x*_, *K*_*y*_, and azimuthal angle Θ:


(26) $$\begin{array}{l} M_{k}=\exp\left[-{\left(\left|\text{K}|-|{\text{K}}_{0}\right|\right)}^{2}/2\Delta_{K}^{2}\right]\exp\left[-{\left(\Theta -\Theta_{0}\right)}^{2}/2\Delta_{\Theta }^{2}\right], \end{array}$$

where **K**_0_ and Θ_0_ run over the locations and azimuthal angles, respectively, of the original **K** modes with lowest spatial frequency, Δ_*K*_ and Δ_Θ_ are the variances of the Gaussian envelope and we set these to $\frac{1}{2}K$ and 20°, respectively, to match experimental observations. The resulting magnitude plot of the two sets of **K** modes for OD and OP map are shown in Figures 12A,B. To approximate the joint structure of OD and OP maps, we set the magnitude and phase of the two K lobes in the OD map in Figure 12A to be identical to the left and right K lobes in the OP map in Figure 12B. Moreover, the top and bottom K lobes in Figure 12B are obtained by rotating the pair of K lobes counterclockwise by 90°. The reconstructed OP-OD map using these two set of **K** is shown in Figure 12C, which resembles the OP-OD maps obtained in experiments (Blasdel, 1992; Bonhoeffer and Grinvald, 1993; Obermayer and Blasdel, 1993). It reproduces the general feature of the OP maps mentioned in Section 2.1.1, including: (i) it has both positive and negative pinwheels, and most of the neighboring pinwheels have opposite signs; (ii) linear zones connect two pinwheel centers; and (iii) most of the pinwheel centers lie in the middle of the OD stripe.

In previous studies (Blasdel, 1992; Niebur and Wörgötter, 1994), the Fourier transform of the OP-OD map of the whole of V1 produced a set of coefficients with near-circular symmetry. Thus, we construct an OP-OD map by expanding the **K** modes more widely in azimuth angle to make the spectrum nearly isotropic, as shown in Figure 12D. Because the annulus is sampled by square pixels, it retains a residual 4-fold symmetry. The resulting reconstructed OP map shown in Figure 12E has more irregular arrangement of OP columns, with no significant preference for horizontal or vertical OD stripes. This figure also underlines the fact that the map is not locally isotropic even if the overall Fourier spectrum is.

### 3.4. Application to OP Maps From a Neural Network Model

In order to analyze the general properties of more realistic OP maps, we perform the same Fourier analysis as on the idealized hypercolumns in previous sections for the OP map generated from a computational neural network model.

**Figure 13:**
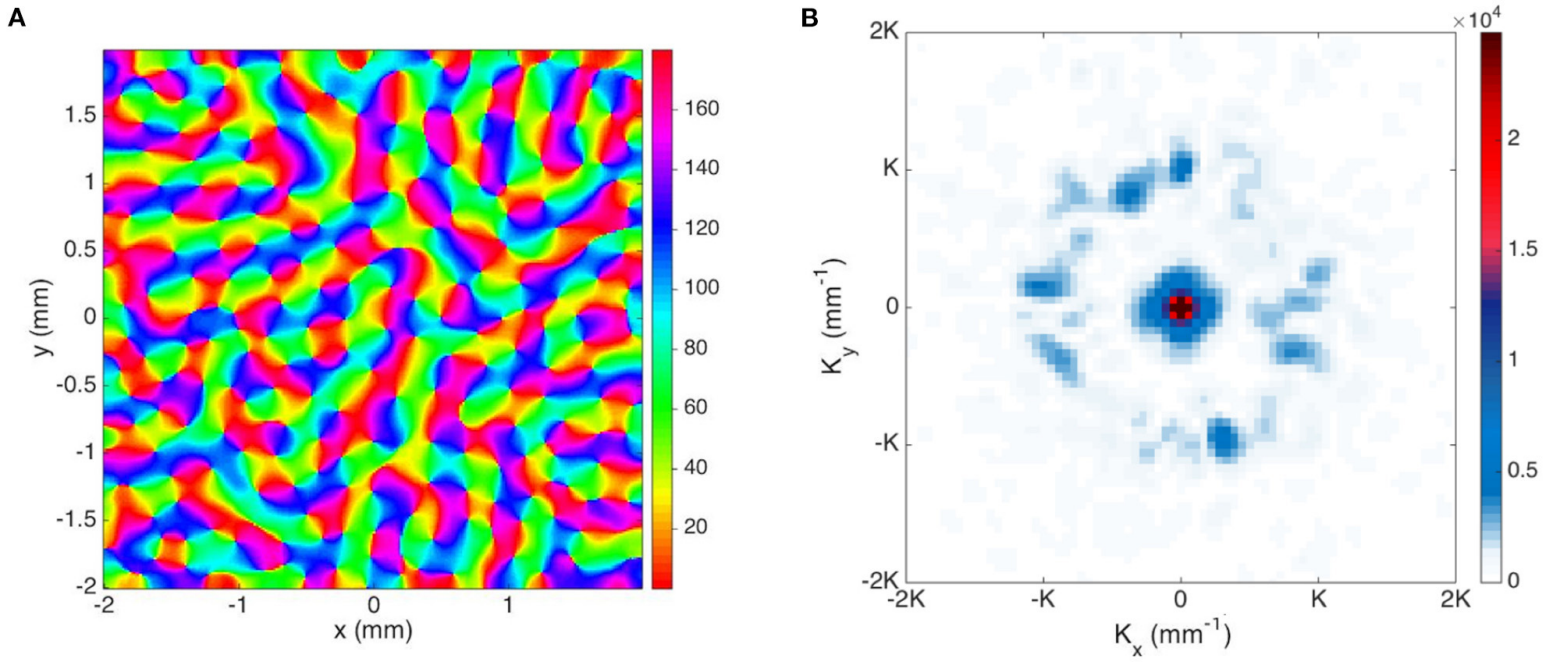
Representations of simulated OP maps. **(A)** OP map generated from GCAL model (Bednar, 2009), and the color bar indicates the OP angle in degrees. **(B)** Magnitude plot of the Fourier coefficients obtained from GCAL OP map. Each pixel-like square represents one spatial mode **K**, and the color bar indicates its magnitude.

The model of V1 we use here is the Gain Control, Adaptation, Laterally Connected (GCAL) model (Stevens et al., 2013), which treats the retina, LGN, and V1 as 2-dimensional sheets, with neurons in each sheet connected topographically. Neurons not only connect to a small group of neurons of the lower level sheet, but also laterally connect to the neurons within the same sheet. A Hebbian learning rule is adopted in the model for updating the connection weights between neurons (Stevens et al., 2013). Figure 13A shows an example output from the GCAL model simulation (Bednar, 2009), and we use this map for further analysis in the Fourier domain.

We process the map in the Fourier domain as follows: (i) Because the 2D discrete Fourier transform implicitly assumes a repeated pattern in both dimensions, the OP has discontinuities at the edges. We minimize these edge effects by doubling the linear dimensions of the array and zero padding the added region. Additionally, we apply a Gaussian window to the map to smooth the edges; (ii) Then we apply the operator O defined in Equation (23), to the resulting map and Fourier transform it.

Figure 13B shows the resulting Fourier coefficients. The dominant terms at |*k*| = *K* are shown in blue. The ring-shaped enhancement near the center arises from zero padding and the large linear size of the overall simulation area, other dominant **K** terms correspond to the periodicity of the hypercolumns and have 4-fold symmetry similar to the pattern in Figure 11A, but with a roughly ±15° spread of **K**. The 4-fold symmetry is most likely at least partly due to a combination of: (i) the artifacts introduced by the approximately square unit cell, and (ii) a structural bias introduced by the square grid on which the GCAL model is simulated. In the simulated map there are roughly ±15° variations, but the Fourier transform still shows signs of 4-fold symmetry resulting from the square simulation boundaries.

## 4. Summary and Conclusion

We present a compact analytic description of an idealized mutually consistent OP-OD map in V1. This includes modeling both the local neuron sensitivity to the stimulus orientation and the weighted projection from nearby neurons, and obtaining a compact Fourier representation of the resulting structure in a form suitable for use in neural field theory. The results and analysis include:

(i) We approximate the periodic OP-OD map in a square grid of hypercolumn with parallel left and right OD stripes with equal width and OP pinwheels with alternating signs. The approximation is idealized but good enough for preserving the basic structure of the OP-OD map.

(ii) We propose a simple approximate AL operator to detect the orientation of the stimulus for local neuron. It is a weighted sum of second order partial derivatives.

(iii) The OP operator reproduces the spatial arrangement of the receptive field of V1 simple cells. It is derived by finding the neuron responses by combining the AL operator with a weighted sum of the projections from neighboring neurons. We optimize the parameters of the operator by controlling the width and angle selectivity of the response tuning curve, and we find that the orientation tuning is only affected significantly by the aspect ratio of the weight function, not the weights of the second order partial derivatives for detecting input orientation in local neuron. The orientation tuning sharpens when we elongate the OP operator along the orientation axis, in accord with experiment (Hubel and Wiesel, 1962a; Lampl et al., 2001).

(iv) We account for the lower OP sensitivity and lower response strength near pinwheel centers by averaging OP over the characteristic microcolumn scale of 40 μm—near centers many different OPs are averaged together, broadening the tuning curve. A Mexican hat function gives a better match to experiment, raising the possibility of using such experimental results to infer the microscopic connectivity profile.

(v) Fourier analysis of a relatively local OD-constrained OP map with the range of ~5 hypercolumns in each direction, were performed to generate two sets of Fourier coefficients for a compact representation of OD and OP, respectively, and especially for use in NFT. The resulting Fourier representation has 2-fold symmetry for OD and 4-fold symmetry for OP due to the idealization of parallel straight OD stripes. Only the **K** modes with lowest frequency are needed to described the spatial structure of OP-OD within a hypercolumn. This simplifies the computational work when we integrate the OP-OD map into NFT to investigate neuronal activity and plasticity in V1. Moreover, if we keep these **K** modes as the basic modes and add extra modes around it using Gaussian envelopes, we could reconstruct a more realistic OP-OD map that is similar to the ones obtained from experiments or computer simulations.

(vi) We also perform Fourier analysis on the irregular OP map generated by the GCAL model. The dominant **K** modes have approximately square symmetry as an artifact of the square grid on which the simulations are done.

Overall, we have focused on modeling the interactions between hypercolumns and local operators for OP feature detection, and have succeeded in obtaining a compact representation of an idealized joint OP-OD map. Notably, the elongated generalized Gaussian operator dominates in determining OP and mutual consistency of OP and OD maps strongly constrains the possible combined maps in hypercolumns, with four pinwheels, not one, required periodic unit of the hypercolumn lattice. The Fourier representation of the idealized structure is sufficiently compact to be used in NFT analyses of V1 structure and activity in future work, with disorder arising from randomness in the drives of cortical plasticity, for example. Moreover, this study can potentially serve as the basis for further modeling of more realistic OP-OD maps on larger scales, and also of other feature maps including the one for direction of motion preference.

## Data Availability Statement

The raw data supporting the conclusions of this article will be made available by the authors, without undue reservation.

## Author Contributions

XL developed the analytical model, performed the numerical computations, produced the figures, and drafted the manuscript. PR contributed to developing the model and revised the manuscript. Both authors contributed to the article and approved the submitted version.

## Funding

This work was supported by the Australian Research Council under Laureate Fellowship grant FL1401000025, Center of Excellence grant CE140100007, and Discovery Project grant DP170101778.

## Conflict of Interest

The authors declare that the research was conducted in the absence of any commercial or financial relationships that could be construed as a potential conflict of interest.

## Publisher's Note

All claims expressed in this article are solely those of the authors and do not necessarily represent those of their affiliated organizations, or those of the publisher, the editors and the reviewers. Any product that may be evaluated in this article, or claim that may be made by its manufacturer, is not guaranteed or endorsed by the publisher.
